# Identification and characterization of microRNAs in the pancreatic fluke *Eurytrema pancreaticum*

**DOI:** 10.1186/1756-3305-6-25

**Published:** 2013-01-25

**Authors:** Min-Jun Xu, Chun-Ren Wang, Si-Yang Huang, Jing-Hua Fu, Dong-Hui Zhou, Qiao-Cheng Chang, Xu Zheng, Xing-Quan Zhu

**Affiliations:** 1 State Key Laboratory of Veterinary Etiological Biology, Key Laboratory of Veterinary Parasitology of Gansu Province, Lanzhou Veterinary Research Institute, Chinese Academy of Agricultural Sciences, Lanzhou, Gansu Province 730046, People’s Republic of China; 2 College of Animal Science and Veterinary Medicine, Heilongjiang Bayi Agricultural University, Daqing, Heilongjiang Province 163319, People’s Republic of China; 3 College of Animal Science, South China Agricultural University, Guangzhou, Guangdong Province, 510642, People’s Republic of China; 4 College of Animal Science and Technology, Yunnan Agricultural University, Kunming, Yunnan Province, 650201, People’s Republic of China

**Keywords:** MicroRNA (miRNA), *Eurytrema pancreaticum*, Profile, Pancreatic fluke, Trematoda

## Abstract

**Background:**

*Eurytrema pancreaticum* is one of the most common flukes, which mainly infects ruminants globally and infects human beings accidentally; causing eurytremiasis that has high veterinary and economic importance. MicroRNAs (miRNAs) are small non-coding RNAs and are now considered as a key mechanism of gene regulation at the post-transcription level.

**Methods:**

We investigated the global miRNA expression profile of *E*. *pancreaticum* adults using next-generation sequencing technology combined with real-time quantitative PCR.

**Results:**

By using the genome of the closely-related species *Schistosoma japonicum* as reference, we obtained 27 miRNA candidates out of 16.45 million raw sequencing reads, with 13 of them found as known miRNAs in *S*. *japonicum* and/or *S*. *mansoni*, and the remaining 14 miRNAs were considered as novel. Five out of the 13 known miRNAs coming from one family named as sja-miR-2, including family members from miR-2a to miR-2e. Targets of 19 miRNAs were successfully predicated out of the 17401 mRNA and EST non-redundant sequences of *S*. *japonicum*. It was found that a significant high number of targets were related to “chch domain-containing protein mitochondrial precursor” (n = 29), “small subunit ribosomal protein s30e” (n = 21), and “insulin-induced gene 1 protein” (n = 9). Besides, “egg protein cp3842” (n = 2), “fumarate hydratase” (n = 2), “ubiquitin-conjugating enzyme” (n = 2), and “sperm-associated antigen 6” (n = 1) were also found as targets of the miRNAs of *E*. *pancreaticum*.

**Conclusions:**

The present study represents the first global characterization of *E*. *pancreaticum* miRNAs, which provides novel resources for a better understanding of the parasite, which, in turn, has implications for the effective control of the disease it causes.

## Background

The pancreatic fluke *Eurytrema pancreaticum* is one of the most common flukes in the pancreas and bile ducts of ruminants [1]. It is closely related to *Schistosoma japonicum*, which has more than 40 species of mammals serving as potential zoonotic reservoirs, which com-plicates parasite transmission dynamics [2]. As a member of the Trematoda, *E*. *pancreaticum* also has a broad range of hosts and mainly infects ruminants, including cattle, buffaloes, pigs, sheep, and goats [3-6]. Some reports indicated that it can accidentally infect humans through dietary habits [7]. *E*. *pancreaticum* infection causes eurytremiasis with high veterinary and economic importance, which is marked by gastrointestinal dis-turbances and progressive weight loss, diarrhea or constipation, and death, with economic losses in meat and milk production [3,8,9]. Eurytremiasis is distributed globally, in South America, Europe and Asia, including countries such as Brazil, China, Japan and Thailand [8,10], and it might be under evaluated due to the current investigating methods [11].

MicroRNAs (miRNAs) are small non-coding RNAs regulating gene expression at the post-transcriptional level and resulting in post-transcriptional repression. miRNAs are conserved in metazoans and can be used as biomarkers [12]. miRNAs were reported in diverse organisms from viruses to mammals [13-15], and are now considered as a key mechanism of gene regulation and are essential for the complex life cycle of pathogenic parasites [13]. The miRNAs of some members of the Trematoda, including *Schistosoma japonicum*, *S*. *mansoni*, *Orientobilharzia turkestanicum*, *Fasciola hepatica* and *F*. *gigantica* have been reported [16-20]. However, there was no miRNAs identified from *E*. *pancreaticum* despite its veterinary and economic importance.

As a member of the Trematoda, *E*. *pancreaticum* may also have miRNAs involved in its gene regulation in the pancreatic fluke. Here we investigated the expression profile of miRNAs and detected potential novel miRNAs in *E*. *pancreaticum* adults. Due to the similarity in morphology, life cycle and modes of transmission among members of the Trematoda [5,21], miRNA profile research in *E*. *pancreaticum* will shed light on the miRNA studies of other species such as *Dicrocoelium dendriticum* and *E*. *coelomaticum*.

## Methods

### Ethics statement

This study was approved by the Animal Ethics Committee of Lanzhou Veterinary Research Institute, Chinese Academy of Agricultural Sciences (Approval No. LVRIAEC2011-006). The sheep from which *E*. *pancreaticum* adults were collected, was handled in accordance with good animal practices required by the Animal Ethics Procedures and Guidelines of the People’s Republic of China.

### Parasites

Adults of *E*. *pancreaticum* were collected from the pancreas of a sheep (Northeast Merino) with a naturally acquired infection in December 2011 in Daqing City, Heilongjiang Province, China. Worms were randomly selected, confirmed as the adult stage with mature eggs based on microscopic examination after staining with carmine [22]. After being washed extensively with sterile physiological saline (37°C) in a sterile beaker, the parasites were transferred to Dulbecco’s modification of Eagle’s medium (DMEM) and incubated at 37°C (10% CO_2_) for 3 h to allow the flukes to regurgitate all the gut contents from their digestive tracts, and then stored at −80°C until use.

### Total RNA and small RNA isolation

Total RNA of ten worms was prepared with Trizol Reagent according to the manufacturer’s protocol (Invitrogen Co. Ltd). Small RNA was prepared as previously [23]. Briefly, RNA fragments of 20–35 bases in length were isolated from 10 μg total RNA with a Novex 15% TBE-Urea gel. These fragments were then reverse transcribed and purified using a 6% TBE PAGE gel. All gels and kits were purchased from Invitrogen Co. Ltd.

### High-throughput sequencing and computational analysis

Samples were sequenced using a Solexa (Illumina) sequencer. Adaptors, low quality reads and reads smaller than 18 nucleotides (nt) were firstly removed from the raw dataset. Rfam database (version 10.1) (http://rfam.sanger.ac.uk/) was searched with BLAST software [24] to remove non-coding RNA, including rRNA, tRNA, snRNA, snoRNA. RepeatMasker (http://www.repeatmasker.org) was used to identify repetitive sequences. Because no publically-available genome is currently accessible for *Eurytrema* spp., the genome of the related schistosome and the closely-related species in genetic distance, *S*. *japonicum* (http://lifecenter.sgst.cn/schistosoma) was used as a reference genome, using the SOAP software [25]. The software Mfold (http://www.bioinfo.rpi.edu/applications/mfold) was used for the prediction of miRNA candidates. The identified miRNA candidates were then searched against the Sanger miRBase (version 17.0) to identify known or conserved miRNAs.

The mRNA and EST data of *S*. *japonicum* were downloaded from the CHGC database (http://www.chgc.sh.cn/japonicum/Resources.html). Potential targets of known miRNAs were predicated with RNAhybrid software [26]. To reduce false-positive results, two extra parameters were performed on the analyzed result: 1) the △△G was set as lower than −25 kcal/mol; 2) P-value was set as ≦0.01. The Gene Ontology (GO, http://www.geneontology.org/) database was used for functional analysis of predicted targets.

### Analysis of novel miRNA transcription

Novel miRNAs were analyzed using a modified stem-loop real-time RT-PCR (ABI PRISM^®^ 7300 Sequence Detection System). All of the primers were synthesized by Shenggong Co, Ltd., China. All reactions were carried out in triplicate. Synthetic *lin4* was used as the endogenous control [27]. The primer pairs were as follows: forward 5^′^-ACACTCCAGCTGGGTCCCTGAGACCTCAAGTG-3^′^ and reverse 5^′^-CTCAACTGGTGTCGTGGAGTCGGCAATTCAGTTGAGTCACACTT-3^′^. The amplification cycle conditions were as follows: 95°C for 5 min, followed by 30 cycles of 95°C for 15 s, 65°C for 15 s, and 72°C for 32 s. The quantification of each miRNA relative to the cel-lin-4 was calculated using the equation: N = 2^-ΔCt^, ΔCt = Ct_miRNA_-Ct_lin4_[28].

## Results

### Profile characteristics of short RNAs

High throughput sequencing yielded 16.45 million raw reads from the total RNA of *E*. *pancreaticum*. After removing low quality reads, adaptors and poly-A sequences, there were 15.35 million reads left with high quality. Length distribution analysis showed that most of the reads were significantly focused on the length of 20 nt with 23.32%; while those around it, including reads of 19, 21 and 22 nt, had only 4.86%, 7.62%, and 7.23%, respectively.

Those representing exons and introns accounted only for a very small percentage of the clean reads (0.18% of unique siRNA), which indicated high integrity of the RNA in the sample. Repeat analysis revealed hundreds of repeat sequences (0.02%), including two types as LINE/RTE:0 (274 reads) and LINE/RTE:1 (558 reads). Other non-coding RNA, including tRNA, rRNA, snRNA and snoRNA, represented a 4.51% of the total.

### Analysis of miRNA profiles

By mapping with the *S*. *japonicum* genome, we obtained 27 miRNA candidates with the precursors having standard stem-loop structures, from the total RNA of the parasite. By matching the miRNA candidates with known Trematoda miRNAs, including 56 *S*. *japonicum* miRNAs (Sja-miR) and 20 *S*. *mansoni* miRNAs (Sma-miR), deposited in the miRBase database, 13 miRNA candidates were previously identified, with 14 novel candidates (Table 1).

**Table 1:** **Known and novel microRNAs (miRNAs) identified from the pancreatic fluke *Eurytrema pancreaticum***

**Name**	**Location at genome**^**a**^	**mfe**^**b**^	**Count**^**c**^	**location**^**d**^	**Sequence**^**e**^
**Known miRNA**					
sja-bantam^f^	SJC_S000254:283545:283639:+	−27.5	3488	3p	TGAGATCGCGATTAAAGCTGG
sja-let-7	SJC_S005824:19533:19622:+	−33.1	50	5p	GGAGGTAGTTCGTTGTGTGGT
sja-miR-2a	SJC_S000054:242652:242727:-	−34.2	4406	3p	TCACAGCCAGTATTGATGAAC
sja-miR-2b	SJC_S000054:242557:242635:-	−33.6	500	3p	TATCACAGCCCTGCTTGGGACAC
sja-miR-2c	SJC_S000102:360645:360725:+	−28.42	62	3p	TATCACAGCCGTGCTTAAGGGC
sja-miR-2d	SJC_S000102:360534:360627:+	−33.1	727	3p	TATCACAGTCCTGCTTAGGTGACG
sja-miR-2e	SJC_S000054:242457:242536:-	−24.7	1721	3p	TATCACAGTCCAAGCTTTGG
sja-miR-71	SJC_S000054:242738:242817:-	−33.01	19048	5p	TGAAAGACGATGGTAGTGAGA
sja-miR-71b	SJC_S000102:360305:360397:+	−36.2	144949	5p	TGAAAGACTTGAGTAGTGAGACGC
sja-miR-124	SJC_S000254:113694:113791:+	−29.8	32	3p	TAAGGCACGCGGTGAATGTCA
sja-miR-8	SJC_S001790:88898:88974:+	−31.5	12	3p	TAATACTGTTAGGTAAAGATGC
sja-miR-2162	SJC_S000471:21744:21822:-	−37.9	13	3p	TATTATGCAACGTTTCACTCT
sja-miR-10^g^	SJC_S000052:310019:310093:+	−23.4	2698/11	5p/3p	AACCCTGTAGACCCGAGTTTG/AAATTCGAGTCTATAAGGA
**Novel miRNA**					
Epa-miR-01	SJC_S000054:242558:242634:-	−31.1	97	3p	TATCACAGCCCTGCTTGGGACA
Epa-miR-02	SJC_S000057:543062:543147:-	−20.4	7	5p	AGAAGGCTGCGTGTTCGGATC
Epa-miR-03	SJC_S000065:176156:176237:+	−22	7	5p	CTGTCTTCTCTCTCATGTTC
Epa-miR-04	SJC_S000083:92590:92686:+	−27.7	16	3p	GTTTGAGACTCCGAATGATG
Epa-miR-05	SJC_S000102:360305:360397:+	−36.2	151	5p	TGAAAGACTTGAGTAGTGAG
Epa-miR-06	SJC_S000102:360535:360626:+	−33.1	111	3p	TATCACAGTCCTGCTTAGGTGAC
Epa-miR-07	SJC_S000102:360645:360725:+	−28.42	62	3p	TATCACAGCCGTGCTTAAGGGC
Epa-miR-08	SJC_S000115:155214:155289:-	−18.2	138	3p	GCTATCTCGTCGATACTGGC
Epa-miR-09	SJC_S000254:113694:113791:+	−29.8	32	3p	TAAGGCACGCGGTGAATGTCA
Epa-miR-10	SJC_S000254:283542:283641:+	−30.7	176	3p	TGAGATCGCGATTAAAGCTGGTT
Epa-miR-11	SJC_S000471:21744:21822:-	−37.9	13	3p	TATTATGCAACGTTTCACTCT
Epa-miR-12	SJC_S001790:88898:88974:+	−31.5	12	3p	TAATACTGTTAGGTAAAGATGC
Epa-miR-13	SJC_S005824:19533:19622:+	−33.1	50	5p	GGAGGTAGTTCGTTGTGTGGT
Epa-miR-14^g^	SJC_S008424:1:71:+	−30.1	33/8	5p/3p	GACGGGGTGGCCGAGTGGTT/CCATTGGGGTTTCCCCGCGT

*Note*: ^a^ location of mature miRNAs at the reference genome of *Schistosoma japonicum*; ^b^ energy of stem-loop structure of miRNA precursors, with unit as Kcal/mol; ^c^ sequencing count; ^d^ location of a mature miRNA at the 3p or 5p arm of its precursor; ^e^ sequence of mature miRNA; ^f^ Known miRNAs matched perfectly with that from *S*. *japonicum* deposited in the miRBase database; ^g^ the only two miRNAs (miR-10, Epa-miR-14) that had mature miRNAs both detected at the 5p and 3p arms of their precursors in the *E*. *pancreaticum*. Blue: the largest family members of miR-2.

Two conserved miRNAs named as bantam and let-7 were found in both *S*. *japonicum* and *S*. *mansoni* miRNAs. The miRNAs bantam and let-7 are conserved miRNAs found in 27 other organisms with miRNAs deposited in the miRBase database, while the let-7 was found distributed in a larger range of 67 other organisms, including invertebrates and vertebrates.

One of the distinguished characteristics of the known miRNAs was that 5 miRNAs were members of one family named miR-2, including members from miR-2a to miR-2e. All of these miRNAs are located at the 3p arm of their precursors. Besides the miR-2 family, another two miRNAs, miR-71 and miR-71b-5p, were also found from one family, which is located on two different scaffolds named as SJC_S000054 and SJC_S000102. Except for miR-10 that had two mature miRNAs located at the 5p and 3p arms of its precusor, all of the other miRNAs had only one mature miRNA found at 5p or 3p arms of their precursors.

**Figure 1:**
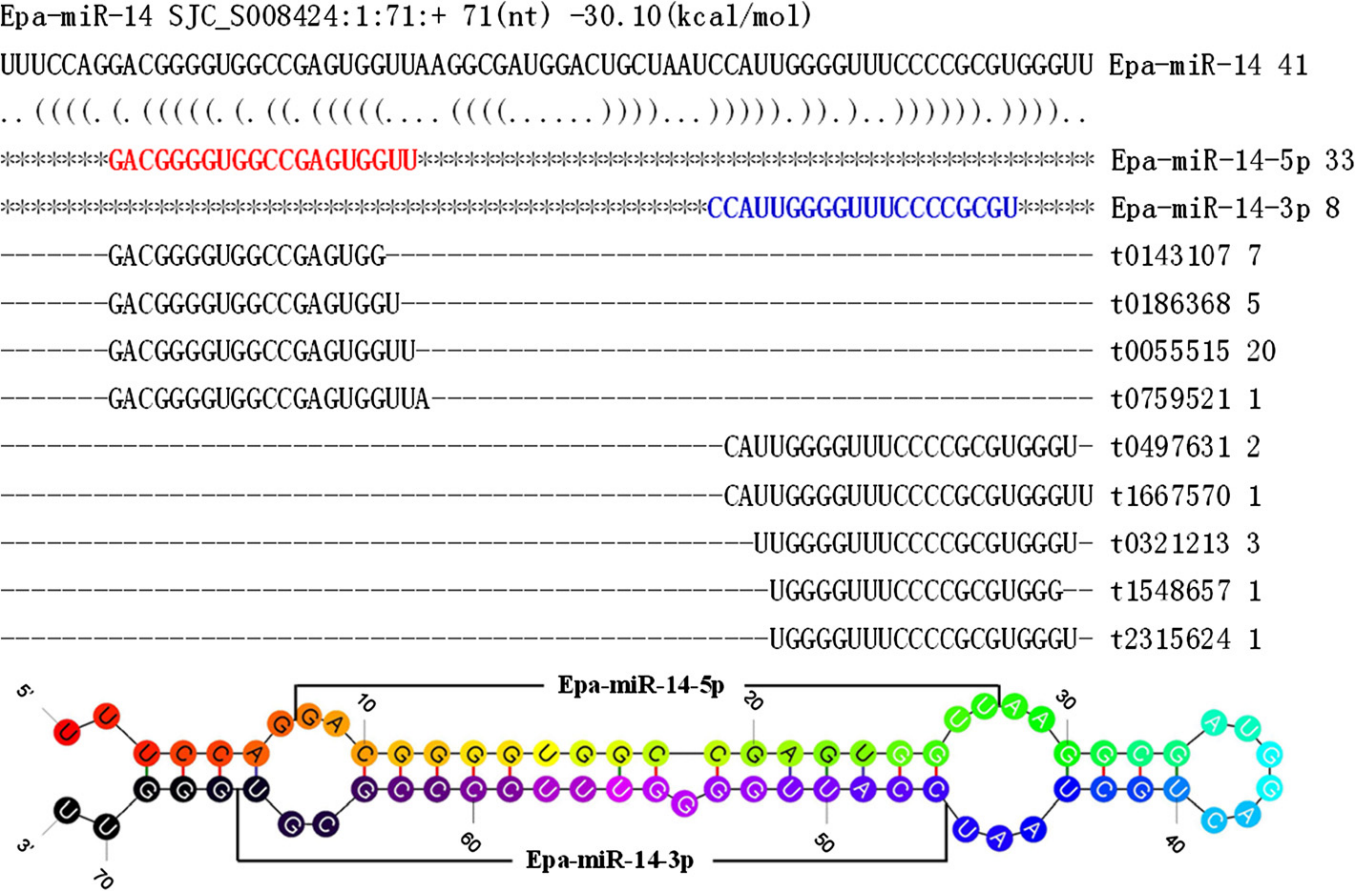
**The stem-loop structure of Epa-miR-14.** The first line including gene location, length of precursor, and energy of stem-loop structure. The mature miRNA in the precursor was shown in red letter (5p) and blue letter (3p). Color from red to black in the stem-loop structure of the miRNA indicated the 5^′^ to 3^′^ direction.

Among the novel miRNAs, 3 miRNAs (Epa-miR-05, Epa-miR-06, and Epa-miR-07) were from one scaffold named as SJC_S000102. Another two miRNAs named Epa-miR-09 and Epa-miR-10 came from another scaffold named as SJC_S000254. Only Epa-miR-14 had both mature miRNAs found at the 5p and 3p of its precursors, and all the others had only one mature miRNA found. The detailed blast information and stem-loop structure of Epa-miR-14 is shown in Figure 1.

### Target prediction and functional analysis

A total of 17,401 mRNA and EST non-redundant sequences of *S*. *japonicum* were downloaded from the CHGC website (See Methods) and used for target prediction for the 27 miRNAs identified in the *E*. *pancreaticum*. Targets of 19 miRNAs were successfully predicted, with numbers ranged from one (Epa-miR-03) to 33 (Epa-miR-01) with 226 in total and an average of 12 (Additional file 1: Table S1).

Out of 226 total targets, 130 targets were successfully illustrated with Blast analysis (Additional file 2: Table S2). Among which, a significant high number of targets were related to “chch domain-containing protein mitochondrial precursor” (n = 29), which were followed by “small subunit ribosomal protein s30e” (n = 21), “insulin-induced gene 1 protein” (n = 9) and “kh domain-RNA-signal transduction-associated protein 1” (n = 7). Besides, “egg protein cp3842” (n = 2), “fumarate hydratase” (n = 2), “ubiquitin-conjugating enzyme” (n = 2), and “sperm-associated antigen 6” (n = 1) were also found as targets of the miRNAs of *E*. *pancreaticum*.

### miRNAs quantification

Four representative novel miRNAs named Epa-miR-05, Epa-miR-06, Epa-miR-08, and Epa-miR-14 were verified using qRT-PCR. The 4 miRNAs had higher sequencing numbers than others and/or had both mature miRNAs on both arms of their precursors. The standard stem-loop structure of Epa-miR-14 precursors is shown in Figure 1.

All the 4 miRNAs could be successfully amplified with qRT-PCR. The relative expression level of Epa-miR-06 (9.06 ± 1.14) was 9.06 fold higher than that of the inner reference gene; the other 2 miRNAs, including Epa-miR-07 and Epa-miR-05 showed similar expression levels of 1.43 ± 0.22 and 1.0 ± 0.19, respectively. For Epa-miR-14, we detected the relative expression level of its mature miRNA at the 5p arm, which was 1.32 ± 0.48.

## Discussion

The objective of this study was to characterize the miRNA profiles of *E*. *pancreaticum*. We obtained 27 miRNA candidates from 16.45 million raw sequencing reads with 13 of them previously known and 14 of them novel. Trematode miRNAs deposited in the miRBase database included 56 *S*. *japonicum* miRNAs and 20 *S*. *mansoni* miRNAs. However, among the 13 known miRNAs only bantam and let-7 were found from both *S*. *japonicum* and *S*. *mansoni*. The remaining 11 known miRNAs were from *S*. *japonicum* miRNAs only. This observation indicated that miRNAs profiles vary between different species in the same class.

miRNAs are known to regulate gene expression at the post-transcriptional level by binding to the 3^′^ UTR of messenger RNA (mRNA) resulting in gene repression, cleavage or destabilization [29,30]. Therefore, miRNAs are essential for the regulation of the complex life cycles of parasites, allowing them to respond to environmental and developmental signals [23,31]. Thus, the novel miRNAs identified in the present study provided novel resources for better understanding of the biology of *E*. *pancreaticum*.

For target prediction and functional analysis, a total of 226 targets with 12 or so in average were obtained for the 19 out of 27 *E*. *pancreaticum* miRNAs. The target number for each gene is low ranging from one to 33, although as many as 17,401 mRNA and EST non-redundant sequences were used. Normally, we found hundreds of miRNA targets for some miRNAs in other species, such as *Ascaris suum*, *A*. *lumbricoides*, and *Toxoplasma gondii* (data not shown). Especially for *Ascaris* spp., where two to three thousands were found for some miRNAs (unpublished observations). The phenomenon of a higher number of targets for one miRNA can also be found in other animals, such as humans, *Caenorhabditis elegans*, and *Drosophilidae* spp. as indicated by popular target predicting websites at present, including TargetScan [32] and Pictar [33]. For the few target phenomenon of *E*. *pancreaticum*, one reason might be that mRNA dataset for target prediction of miRNAs of *E*. *pancreaticum* was from another trematode, *S*. *japonicum*, instead of the parasite itself, for which the transcriptome data is not available at present; another reason might be that this is a specific character of the miRNAs of *E*. *pancreaticum*. However, more experimental information is needed to verify these possibilities.

Of the predicted targets, a significant high number of targets were related to the “chch domain-containing protein” (n = 29). The “chch domain” is also called “churchill domain”, belongs to a zinc finger transcriptional activator. It was reported that the protein regulated the transition between gastrulation and neurulation, and regulates cell ingression [34]. Besides, it was interesting to find that both “egg protein” (n = 2) and “sperm-associated antigen” (n = 1) were found as targets of the miRNAs of *E*. *pancreaticum*.

## Conclusions

In the present study, the miRNA profiles of the pancreatic fluke *E*. *pancreaticum* were investigated and 27 miRNAs were identified from the pancreatic fluke. Furthermore, we also investigated the potential targets and their functions of 19 of the 27 miRNAs. The present study represented the first global characterization of *E*. *pancreaticum* miRNAs, which provides novel resources for better understanding of the biology of the parasite, which, in turn, has implications for the effective control of the disease it causes.

## Competing interests

The authors declare that they have no competing interests.

## Authors’ contributions

XQZ and MJX conceived and designed the study, and critically revised the manuscript. MJX, CRW and JHF performed the experiments, analyzed the data and drafted the manuscript. SYH, DHZ, QCC and XZ helped in study design, study implementation and manuscript revision. All authors read and approved the final manuscript.

## Supplementary Material

Additional file 1: Table S1 Predicated miRNA targets of *Eurytrema pancreaticum*. Click here for file

Additional file 2: Table S2 Blast analysis of miRNA targets of *Eurytrema pancreaticum*. Click here for file
