# Supplementary material for: Identification and characterization of microRNAs in the pancreatic fluke Eurytrema pancreaticum
Source: Parasit Vectors. 2013 Jan 25;6:25. doi: 10.1186/1756-3305-6-25 (PMC3621695; doi:10.1186/1756-3305-6-25)
Supplement: Additional file 1: Table S1 — Predicated miRNA targets of Eurytrema pancreaticum. [file 1756-3305-6-25-S1.docx]

**Table S1. Predicated miRNA targets of *Eurytrema pancreaticum***

| **Name** | **Length** | **Total target number** | **Best matched Target** | **Description of the target** | **Mfe** |
| --- | --- | --- | --- | --- | --- |
| Known miRNA | |  |  |  |  |
| sja-let-7-5p | 21 | 6 | SJFCA0067 | DNMAP1 | -32.9 |
| sja-miR-10-5p | 21 | 31 | SJFCE4621 | N/A | -31.2 |
| sja-miR-124-3p | 21 | 7 | SJFCA3849.001 | rps-26 | -33.5 |
| sja-miR-2b-3p | 23 | 26 | SJFCE2369.001 | Fh1 | -37.8 |
| sja-miR-2c-3p | 22 | 2 | SJFCE2510.001 | Sperm-associated antigen 6 | -36.8 |
| sja-miR-2d-3p | 24 | 2 | SJFCE3096.002 | Uev1A | -32.2 |
| sja-miR-8-3p | 22 | 21 | SJFCE3828.001 | small subunit ribosomal protein S30e; | -28.5 |
| Novel miRNA | |  |  |  |  |
| Epa-miR-01-3p | 22 | 33 | SJFCE2369.001 | Fh1 | -37.3 |
| Epa-miR-02-5p | 21 | 15 | SJFCE3560.007 | signal transduction-associated protein 1; | -32 |
| Epa-miR-03-5p | 20 | 1 | SJFCA4312 | N/A | -29.4 |
| Epa-miR-06-3p | 23 | 2 | SJFCE3096.002 | Uev1A | -30.9 |
| Epa-miR-07-3p | 22 | 2 | SJFCE2510.001 | Sperm-associated antigen 6; | -36.8 |
| Epa-miR-08-3p | 20 | 2 | SJFCA0559 | N/A | -38.4 |
| Epa-miR-09-3p | 21 | 7 | SJFCA3849.001 | rps-26 | -33.5 |
| Epa-miR-10-3p | 23 | 11 | SJFCE3730.001 | hypothetical protein; | -28.3 |
| Epa-miR-12-3p | 22 | 21 | SJFCE3828.001 | small subunit ribosomal protein S30e; | -28.5 |
| Epa-miR-13-5p | 21 | 6 | SJFCA0067 | DNMAP1 | -32.9 |
| Epa-miR-14-3p | 20 | 4 | SJFCE1649 | VAMP2 | -34.5 |
| Epa-miR-14-5p | 20 | 27 | SJFCE1354 | TIM21-like protein | -36.5 |
