# Supplementary material for: Identification and characterization of microRNAs in the pancreatic fluke Eurytrema pancreaticum
Source: Parasit Vectors. 2013 Jan 25;6:25. doi: 10.1186/1756-3305-6-25 (PMC3621695; doi:10.1186/1756-3305-6-25)
Supplement: Additional file 2: Table S2 — Blast analysis of miRNA targets of Eurytrema pancreaticum. [file 1756-3305-6-25-S2.docx]

**Table S2. Blast analysis of miRNA targets of *Eurytrema pancreaticum***

| **Sequence name** | **Sequence description** | **Sequence length** | **Hit ACC** | **E-Value** | **Similarity** | **Score** | **Alignment length** | **Positives** |
| --- | --- | --- | --- | --- | --- | --- | --- | --- |
| SJFCA0067 | DNA methyltransferase 1-associated protein 1 (dnmt1-associated protein 1) | 485 | CAX69350 | 7.94E-81 | 100 | 245.358 | 116 | 116 |
| SJFCA0201 | zinc finger protein 207 | 1140 | CAX69417 | 0 | 100 | 607.831 | 334 | 334 |
| SJFCA4035 | sjchgc06879 protein | 323 | AAX25234 | 8.77E-19 | 68 | 83.9593 | 89 | 61 |
| SJFCA4069 | hypothetical protein | 655 | CAX72711 | 1.25E-20 | 100 | 90.5077 | 42 | 42 |
| SJFCA3828.021 | small subunit ribosomal protein s30e | 520 | ABF19508 | 3.28E-90 | 100 | 270.396 | 135 | 135 |
| SJFCA3859.007 | chch domain-containing protein mitochondrial precursor | 509 | AAW26101 | 3.76E-35 | 100 | 129.413 | 106 | 106 |
| SJFCA3849.001 | ribosomal small subunit | 1054 | CAX71860 | 1.16E-52 | 100 | 179.104 | 81 | 81 |
| SJFCA3859.014 | chch domain-containing protein mitochondrial precursor | 519 | AAW26101 | 4.23E-35 | 100 | 129.413 | 106 | 106 |
| SJFCA3096.001 | ubiquitin-conjugating enzyme | 624 | CAX70253 | 1.83E-107 | 100 | 316.235 | 149 | 149 |
| SJFCA0333 | signal transduction protein lnk-related | 441 | CAX69501 | 9.96E-74 | 100 | 226.483 | 109 | 109 |
| SJFCA3828.008 | small subunit ribosomal protein s30e | 476 | ABF19508 | 1.84E-90 | 100 | 270.396 | 135 | 135 |
| SJFCA0348 | transmembrane protein 115-like | 1423 | XP_002571754 | 8.74E-108 | 87 | 218.779 | 174 | 153 |
| SJFCA3859.006 | chch domain-containing protein mitochondrial precursor | 502 | CAX71920 | 2.53E-38 | 100 | 137.502 | 106 | 106 |
| SJFCA3859.011 | chch domain-containing protein mitochondrial precursor | 517 | AAW26101 | 4.09E-35 | 100 | 129.413 | 106 | 106 |
| SJFCA3887.033 | cathepsin a | 191 | CAX72171 | 1.85E-24 | 100 | 101.293 | 47 | 47 |
| SJFCA3859.017 | chch domain-containing protein mitochondrial precursor | 535 | AAW26101 | 4.96E-35 | 100 | 129.413 | 106 | 106 |
| SJFCA3560.004 | kh domain-RNA-signal transduction-associated protein 1 | 1198 | CAX70846 | 0 | 100 | 612.453 | 306 | 306 |
| SJFCA0495 | u6 snRNA-associated sm-like protein lsm5 | 371 | CAX69600 | 1.95E-54 | 100 | 175.637 | 87 | 87 |
| SJFCA3732.011 | hypothetical protein | 422 | CAX76868 | 1.12E-30 | 100 | 114.775 | 60 | 60 |
| SJFCA3614.004 | insulin-induced gene 1 protein | 916 | CAX76199 | 4.24E-131 | 100 | 382.489 | 206 | 206 |
| SJFCA3335.005 | sjchgc06375 protein | 331 | AAX24720 | 1.63E-58 | 96 | 189.504 | 96 | 93 |
| SJFCA3004.003 | 60s ribosomal protein l7 | 1222 | CAX74658 | 3.02E-126 | 100 | 243.817 | 125 | 125 |
| SJFCA3828.009 | small subunit ribosomal protein s30e | 476 | ABF19508 | 1.82E-90 | 100 | 270.396 | 135 | 135 |
| SJFCA3859.008 | chch domain-containing protein mitochondrial precursor | 512 | AAW26101 | 3.88E-35 | 100 | 129.413 | 106 | 106 |
| SJFCA3859.018 | chch domain-containing protein mitochondrial precursor | 535 | CAX71920 | 3.64E-38 | 100 | 137.502 | 106 | 106 |
| SJFCA3859.019 | chch domain-containing protein mitochondrial precursor | 535 | AAW26101 | 4.96E-35 | 100 | 129.413 | 106 | 106 |
| SJFCA3859.009 | chch domain-containing protein mitochondrial precursor | 515 | AAW26101 | 3.96E-35 | 100 | 129.413 | 106 | 106 |
| SJFCA3859.020 | chch domain-containing protein mitochondrial precursor | 535 | AAW26101 | 4.96E-35 | 100 | 129.413 | 106 | 106 |
| SJFCA4312 | sjchgc02645 protein | 586 | AAW27766 | 1.24E-13 | 56 | 73.559 | 106 | 60 |
| SJFCA3859.005 | chch domain-containing protein mitochondrial precursor | 501 | AAW26101 | 3.48E-35 | 100 | 129.413 | 106 | 106 |
| SJFCE3730.001 | hypothetical protein [*Schistosoma japonicum*] | 236 | CAX76844 | 1.11E-14 | 100 | 70.8626 | 49 | 49 |
| SJFCE3730.002 | hypothetical protein [*Schistosoma japonicum*] | 236 | CAX76844 | 1.13E-14 | 100 | 70.8626 | 49 | 49 |
| SJFCE2369.002 | fumarate hydratase | 1700 | CAX73952 | 0 | 100 | 968.763 | 485 | 485 |
| SJFCE2369.001 | fumarate hydratase | 1684 | CAX73951 | 0 | 100 | 968.763 | 485 | 485 |
| SJFCE3732.004 | hypothetical protein | 277 | CAX76868 | 2.04E-31 | 100 | 114.775 | 60 | 60 |
| SJFCE3859.015 | chch domain-containing protein mitochondrial precursor | 519 | AAW26101 | 4.23E-35 | 100 | 129.413 | 106 | 106 |
| SJFCE3732.013 | hypothetical protein | 493 | CAX76868 | 2.33E-30 | 100 | 114.775 | 60 | 60 |
| SJFCE3732.003 | hypothetical protein | 272 | CAX76868 | 1.87E-31 | 100 | 114.775 | 60 | 60 |
| SJFCE3730.013 | hypothetical protein [*Schistosoma japonicum*] | 289 | CAX76850 | 2.93E-04 | 91 | 43.5134 | 37 | 34 |
| SJFCE3552.005 | cysteine-rich protein 1 | 407 | AAX30636 | 2.93E-41 | 100 | 142.124 | 75 | 75 |
| SJFCE3859.028 | chch domain-containing protein mitochondrial precursor | 549 | AAW26101 | 5.63E-35 | 100 | 129.413 | 106 | 106 |
| SJFCE1354 | tim21-like mitochondrial precursor | 782 | CAX73241 | 1.86E-126 | 100 | 369.392 | 221 | 221 |
| SJFCE3828.014 | small subunit ribosomal protein s30e | 486 | ABF19508 | 2.07E-90 | 100 | 270.396 | 135 | 135 |
| SJFCE3828.001 | small subunit ribosomal protein s30e | 472 | ABF19508 | 1.68E-90 | 100 | 270.396 | 135 | 135 |
| SJFCE3732.001 | hypothetical protein | 268 | CAX76867 | 8.98E-32 | 100 | 115.546 | 60 | 60 |
| SJFCE3828.020 | small subunit ribosomal protein s30e | 501 | ABF19508 | 2.49E-90 | 100 | 270.396 | 135 | 135 |
| SJFCE3671.002 | small nuclear ribonucleoprotein-associated protein b | 744 | CAX76474 | 3.33E-131 | 100 | 381.333 | 231 | 231 |
| SJFCE3614.008 | insulin-induced gene 1 protein | 884 | CAX76199 | 2.62E-129 | 100 | 377.481 | 203 | 203 |
| SJFCE3859.013 | chch domain-containing protein mitochondrial precursor | 518 | AAW26101 | 4.09E-35 | 100 | 129.413 | 106 | 106 |
| SJFCE3828.005 | small subunit ribosomal protein s30e | 474 | ABF19508 | 1.84E-90 | 100 | 270.396 | 135 | 135 |
| SJFCE3614.005 | insulin-induced gene 1 protein | 916 | CAX76199 | 4.24E-131 | 100 | 382.489 | 206 | 206 |
| SJFCE3732.012 | hypothetical protein | 471 | CAX76868 | 1.87E-30 | 100 | 114.775 | 60 | 60 |
| SJFCE3905.010 | egg protein cp3842 | 791 | CAX79490 | 1.94E-127 | 100 | 371.318 | 203 | 203 |
| SJFCE3732.002 | hypothetical protein | 268 | CAX76868 | 1.80E-31 | 100 | 114.775 | 60 | 60 |
| SJFCE3059.001 | Ankyrin, domain-containing protein [*Schistosoma japonicum*] | 911 | CAX74741 | 3.10E-124 | 100 | 364.77 | 180 | 180 |
| SJFCE3730.011 | hypothetical protein [*Schistosoma japonicum*] | 247 | CAX76844 | 1.27E-14 | 100 | 70.8626 | 49 | 49 |
| SJFCE3004.001 | 60s ribosomal protein l7 | 862 | CAX74658 | 2.57E-162 | 99 | 462.611 | 248 | 247 |
| SJFCE3828.012 | small subunit ribosomal protein s30e | 484 | ABF19508 | 2.05E-90 | 100 | 270.396 | 135 | 135 |
| SJFCE3614.007 | insulin-induced gene 1 protein | 911 | CAX76202 | 3.11E-97 | 100 | 293.893 | 143 | 143 |
| SJFCE3552.004 | cysteine-rich protein 1 | 421 | AAX30636 | 3.50E-41 | 100 | 142.124 | 75 | 75 |
| SJFCE4888 | b9 protein domain 1 | 808 | AAX28461 | 1.75E-110 | 100 | 245.743 | 123 | 123 |
| SJFCE3004.002 | 60s ribosomal protein l7 | 839 | CAX74658 | 9.11E-175 | 100 | 493.812 | 248 | 248 |
| SJFCE3859.021 | chch domain-containing protein mitochondrial precursor | 535 | AAW26101 | 4.96E-35 | 100 | 129.413 | 106 | 106 |
| SJFCE2510.001 | sperm-associated antigen 6 | 1891 | CAX74092 | 0 | 100 | 925.235 | 496 | 496 |
| SJFCE3859.022 | chch domain-containing protein mitochondrial precursor | 537 | AAW26101 | 1.95E-23 | 100 | 75.8702 | 35 | 35 |
| SJFCE3828.019 | small subunit ribosomal protein s30e | 500 | ABF19508 | 2.52E-90 | 100 | 270.396 | 135 | 135 |
| SJFCE3560.007 | kh domain- RNA- signal transduction-associated protein 1 | 1136 | CAX75963 | 0 | 100 | 634.41 | 312 | 312 |
| SJFCE3732.008 | hypothetical protein | 305 | CAX76868 | 2.85E-31 | 100 | 114.775 | 60 | 60 |
| SJFCE2988.002 | dual specificity phosphatase 11 (RNA RNP complex 1-interacting) | 511 | CAX74638 | 4.83E-52 | 100 | 171.014 | 74 | 74 |
| SJFCE3732.005 | hypothetical protein | 278 | CAX76868 | 2.04E-31 | 100 | 114.775 | 60 | 60 |
| SJFCE1564 | protein kinase | 1553 | CAX73354 | 0 | 100 | 682.559 | 342 | 342 |
| SJFCE3730.005 | hypothetical protein [*Schistosoma japonicum*] | 236 | CAX76848 | 5.62E-15 | 100 | 71.633 | 49 | 49 |
| SJFCE3828.015 | small subunit ribosomal protein s30e | 486 | ABF19508 | 2.05E-90 | 100 | 270.396 | 135 | 135 |
| SJFCE3560.006 | kh domain- RNA- signal transduction-associated protein 1 | 1167 | CAX75963 | 0 | 100 | 634.41 | 312 | 312 |
| SJFCE3828.006 | small subunit ribosomal protein s30e | 475 | CAX77936 | 1.82E-90 | 100 | 270.396 | 135 | 135 |
| SJFCE3284.002 | hypothetical protein | 320 | CAX75165 | 3.34E-10 | 100 | 60.077 | 50 | 50 |
| SJFCE1619 | sjchgc08542 protein | 271 | AAX25193 | 5.06E-43 | 100 | 147.902 | 83 | 83 |
| SJFCE3859.024 | chch domain-containing protein mitochondrial precursor | 539 | AAW26101 | 5.18E-35 | 100 | 129.413 | 106 | 106 |
| SJFCE3732.009 | hypothetical protein | 317 | CAX76868 | 3.26E-31 | 100 | 114.775 | 60 | 60 |
| SJFCE1649 | sjchgc01984 protein | 926 | AAP05935 | 1.10E-70 | 100 | 226.868 | 160 | 160 |
| SJFCE3614.009 | insulin-induced gene 1 protein | 879 | CAX76198 | 1.38E-122 | 99 | 360.147 | 177 | 176 |
| SJFCE3560.001 | kh domain- RNA- signal transduction-associated protein 1 | 1221 | CAX75963 | 0 | 100 | 634.41 | 312 | 312 |
| SJFCE3828.016 | small subunit ribosomal protein s30e | 486 | ABF19508 | 2.05E-90 | 100 | 270.396 | 135 | 135 |
| SJFCE3730.007 | hypothetical protein [*Schistosoma japonicum*] | 237 | CAX76850 | 2.31E-08 | 100 | 54.299 | 49 | 49 |
| SJFCE3730.010 | hypothetical protein [*Schistosoma japonicum*] | 243 | CAX76852 | 1.19E-14 | 100 | 70.8626 | 49 | 49 |
| SJFCE1703 | mitochondrial ribosomal protein 63 | 444 | AAW25385 | 2.74E-77 | 97 | 235.728 | 115 | 112 |
| SJFCE3165.001 | sjchgc01019 protein | 650 | CAX74934 | 1.23E-125 | 100 | 364.77 | 202 | 202 |
| SJFCE3828.011 | small subunit ribosomal protein s30e | 480 | CAX77939 | 9.41E-91 | 100 | 271.166 | 135 | 135 |
| SJFCE3552.006 | cysteine-rich protein 1 | 406 | AAX30636 | 2.93E-41 | 100 | 142.124 | 75 | 75 |
| SJFCE3096.002 | ubiquitin-conjugating enzyme | 595 | CAX74797 | 4.74E-115 | 100 | 335.495 | 161 | 161 |
| SJFCE3828.018 | small subunit ribosomal protein s30e | 495 | ABF19508 | 2.33E-90 | 100 | 270.396 | 135 | 135 |
| SJFCE3560.005 | kh domain- RNA- signal transduction-associated protein 1 | 1194 | CAX75963 | 0 | 100 | 634.41 | 312 | 312 |
| SJFCE3859.001 | chch domain-containing protein mitochondrial precursor | 475 | CAX71920 | 1.80E-38 | 100 | 137.502 | 106 | 106 |
| SJFCE3730.012 | hypothetical protein [*Schistosoma japonicum*] | 266 | CAX76844 | 8.50E-14 | 100 | 68.9366 | 48 | 48 |
| SJFCE3915.011 | ribosomal protein l30 | 409 | CAX72575 | 2.69E-68 | 100 | 212.616 | 105 | 105 |
| SJFCE3614.002 | insulin-induced gene 1 protein | 932 | CAX76198 | 3.62E-127 | 100 | 372.474 | 200 | 200 |
| SJFCE3828.013 | small subunit ribosomal protein s30e | 484 | CAX77941 | 8.73E-68 | 100 | 211.846 | 106 | 106 |
| SJFCE3732.006 | hypothetical protein | 290 | CAX76868 | 2.37E-31 | 100 | 114.775 | 60 | 60 |
| SJFCE3859.010 | chch domain-containing protein mitochondrial precursor | 516 | AAW26101 | 3.96E-35 | 100 | 129.413 | 106 | 106 |
| SJFCE3828.017 | small subunit ribosomal protein s30e | 486 | ABF19508 | 2.07E-90 | 100 | 270.396 | 135 | 135 |
| SJFCE3730.008 | hypothetical protein [*Schistosoma japonicum*] | 237 | CAX76852 | 1.55E-24 | 95 | 96.2857 | 49 | 47 |
| SJFCE3552.007 | cysteine-rich protein 1 | 406 | AAX30636 | 2.93E-41 | 100 | 142.124 | 75 | 75 |
| SJFCE3828.010 | small subunit ribosomal protein s30e | 476 | ABF19508 | 1.84E-90 | 100 | 270.396 | 135 | 135 |
| SJFCE3730.006 | hypothetical protein [*Schistosoma japonicum*] | 236 | CAX76849 | 6.13E-14 | 100 | 68.9366 | 49 | 49 |
| SJFCE3828.004 | small subunit ribosomal protein s30e | 474 | ABF19508 | 1.75E-90 | 100 | 270.396 | 135 | 135 |
| SJFCE3859.012 | chch domain-containing protein mitochondrial precursor | 517 | CAX71920 | 2.99E-38 | 100 | 137.502 | 106 | 106 |
| SJFCE3614.006 | insulin-induced gene 1 protein | 916 | CAX76199 | 4.29E-131 | 100 | 382.489 | 206 | 206 |
| SJFCE3859.002 | chch domain-containing protein mitochondrial precursor | 476 | AAW26101 | 2.58E-35 | 100 | 129.413 | 106 | 106 |
| SJFCE1902 | tbc1 domain family member 8 (vascular rab-gap tbc-containing protein) | 1117 | CAX73536 | 0 | 100 | 583.178 | 285 | 285 |
| SJFCE3859.025 | chch domain-containing protein mitochondrial precursor | 539 | AAW26101 | 5.12E-35 | 100 | 129.413 | 106 | 106 |
| SJFCE3560.003 | kh domain- RNA- signal transduction-associated protein 1 | 1205 | CAX75963 | 0 | 100 | 634.41 | 312 | 312 |
| SJFCE3817.006 | immunogenic miracidial antigen 8i | 513 | CAX77765 | 1.22E-26 | 100 | 105.916 | 79 | 79 |
| SJFCE3614.003 | insulin-induced gene 1 protein | 918 | CAX76199 | 4.40E-131 | 100 | 382.489 | 206 | 206 |
| SJFCE3859.026 | chch domain-containing protein mitochondrial precursor | 540 | AAW26101 | 5.28E-35 | 100 | 129.413 | 106 | 106 |
| SJFCE3859.027 | chch domain-containing protein mitochondrial precursor | 541 | AAW26101 | 5.28E-35 | 100 | 129.413 | 106 | 106 |
| SJFCE3859.023 | chch domain-containing protein mitochondrial precursor | 538 | AAW26101 | 5.18E-35 | 100 | 129.413 | 106 | 106 |
| SJFCE3828.002 | small subunit ribosomal protein s30e | 473 | ABF19508 | 1.75E-90 | 100 | 270.396 | 135 | 135 |
| SJFCE3828.007 | small subunit ribosomal protein s30e | 475 | ABF19508 | 1.84E-90 | 100 | 270.396 | 135 | 135 |
| SJFCE3109.004 | signal sequence delta | 641 | AAW25112 | 1.64E-110 | 100 | 324.709 | 163 | 163 |
| SJFCE3859.016 | chch domain-containing protein mitochondrial precursor | 532 | AAW26101 | 4.86E-35 | 100 | 129.413 | 106 | 106 |
| SJFCE3905.013 | egg protein cp3842 | 791 | CAX79490 | 1.97E-127 | 100 | 371.318 | 203 | 203 |
| SJFCE3828.003 | small subunit ribosomal protein s30e | 473 | CAX77933 | 4.72E-87 | 100 | 261.536 | 131 | 131 |
| SJFCE3859.003 | chch domain-containing protein mitochondrial precursor | 477 | AAW26101 | 1.22E-27 | 100 | 98.5969 | 45 | 45 |
| SJFCE3732.010 | hypothetical protein | 397 | CAX76868 | 8.17E-31 | 100 | 114.775 | 60 | 60 |
| SJFCE3859.029 | chch domain-containing protein mitochondrial precursor | 551 | AAW26101 | 5.81E-35 | 100 | 129.413 | 106 | 106 |
| SJFCE3859.004 | chch domain-containing protein mitochondrial precursor | 497 | AAW26101 | 3.26E-35 | 100 | 129.413 | 106 | 106 |
| SJFCE3732.007 | hypothetical protein | 304 | CAX76868 | 2.71E-31 | 100 | 114.775 | 60 | 60 |
| SJFCE3730.009 | hypothetical protein [*Schistosoma japonicum*] | 237 | CAX76850 | 2.31E-08 | 100 | 54.299 | 49 | 49 |
| SJFCE3614.001 | insulin-induced gene 1 protein | 955 | CAX76199 | 1.31E-99 | 100 | 287.345 | 140 | 140 |
| SJFCE3560.002 | kh domain- RNA- signal transduction-associated protein 1 | 1221 | CAX75963 | 0 | 100 | 634.41 | 312 | 312 |
